# Flower Services and Hospitals

**Published:** 1887-07-16

**Authors:** 


### FLOWER-SERVICES AND HOSPITALS.

Mr. P. Michelli, the Secretary of the Seamen's Hospital Society (late Dreadnought), Greenwich, writes:—

" Within the last few days we have received a large quantity of flowers from the following places of worship:—Blackheath Congregational Church, per S. M. Osmond, Esq.; New Street Deptford Chapel; Holy Trinity Church, Lee; Burnt-ash Hill Church; Bourton-on-Hill Church, per Rev. R. Mitford Taylor, Jun.; St. James's, Hatcham. Such a profusion of flowers is most welcome to our sick sailors; but one almost wishes that they had been spread over a longer period."

